# *It could be viral but you don’t know, you have not diagnosed it*: health worker challenges in managing non-malaria paediatric fevers in the low transmission area of Mbarara District, Uganda

**DOI:** 10.1186/s12936-016-1257-y

**Published:** 2016-04-11

**Authors:** Emily White Johansson, Freddy Eric Kitutu, Chrispus Mayora, Phyllis Awor, Stefan Swartling Peterson, Henry Wamani, Helena Hildenwall

**Affiliations:** Department of Women’s and Children’s Health, Uppsala University, Uppsala, Sweden; Makerere University School of Public Health, College of Health Sciences, Kampala, Uganda; School of Public Health, University of Witwatersrand, Johannesburg, South Africa; Department of Global Public Health and Primary Care, University of Bergen, Bergen, Norway; Karolinska Institute, Global Health–Health Systems and Policy Research Group, Stockholm, Sweden

**Keywords:** Uganda, Child health, Malaria, Fever, Diagnosis, Qualitative, Point-of-care diagnostics

## Abstract

**Background:**

In 2012, Uganda initiated nationwide deployment of malaria rapid diagnostic tests (RDT) as recommended by national guidelines. Yet growing concerns about RDT non-compliance in various settings have spurred calls to deploy RDT as part of enhanced support packages. An understanding of how health workers currently manage non-malaria fevers, particularly for children, and challenges faced in this work should also inform efforts.

**Methods:**

A qualitative study was conducted in the low transmission area of Mbarara District (Uganda). In-depth interviews with 20 health workers at lower level clinics focused on RDT perceptions, strategies to differentiate non-malaria paediatric fevers, influences on clinical decisions, desires for additional diagnostics, and any challenges in this work. Seven focus group discussions were conducted with caregivers of children under 5 years of age in facility catchment areas to elucidate their RDT perceptions, understandings of non-malaria paediatric fevers and treatment preferences. Data were extracted into meaning units to inform codes and themes in order to describe response patterns using a latent content analysis approach.

**Results:**

Differential diagnosis strategies included studying fever patterns, taking histories, assessing symptoms, and analysing other factors such as a child’s age or home environment. If no alternative cause was found, malaria treatment was reportedly often prescribed despite a negative result. Other reasons for malaria over-treatment stemmed from RDT perceptions, system constraints and provider-client interactions. RDT perceptions included mistrust driven largely by expectations of false negative results due to low parasite/antigen loads, previous anti-malarial treatment or test detection of only one species. System constraints included poor referral systems, working alone without opportunity to confer on difficult cases, and lacking skills and/or tools for differential diagnosis. Provider-client interactions included reported caregiver RDT mistrust, demand for certain drugs and desire to know the ‘exact’ disease cause if not malaria. Many health workers expressed uncertainty about how to manage non-malaria paediatric fevers, feared doing wrong and patient death, worried caregivers would lose trust, or felt unsatisfied without a clear diagnosis.

**Conclusions:**

Enhanced support is needed to improve RDT adoption at lower level clinics that focuses on empowering providers to successfully manage non-severe, non-malaria paediatric fevers without referral. This includes building trust in negative results, reinforcing integrated care initiatives (e.g., integrated management of childhood illness) and fostering communities of practice according to the diffusion of innovations theory.

## Background

Presumptive malaria treatment of all febrile children has long been promoted in malaria-endemic African settings without adequate diagnostics [1]. In 2010 however, the World Health Organization (WHO) revised guidelines to recommend parasitological diagnosis of all suspected malaria cases and treatment based on test results [2]. Uganda subsequently revised national malaria treatment guidelines in 2012 and began nationwide deployment of malaria rapid diagnostic tests (RDT) in order to achieve universal diagnosis goals [3].

This policy shift has great potential to improve rational drug use and quality fever management by excluding malaria as the fever cause and prompting health workers to further assess and treat other conditions [4]. Yet many studies indicate continued inappropriate management of acute febrile illnesses even after RDT introduction [5–7]. Health workers commonly prescribe anti-malarial drugs to febrile patients despite a negative test result [6, 7]. In studies where these drugs were largely restricted to positive cases, some research indicates widespread antibiotic prescriptions for test-negative patients [8, 9].

Growing concerns about RDT non-compliance have spurred calls to deploy RDT with enhanced support packages and as part of integrated fever management protocols, notably integrated management of childhood illness (IMCI) for sick children [10]. This important effort should also be grounded in a broad understanding of how health workers currently manage non-malaria fevers at lower level facilities, their *own* desires for additional support or diagnostics, and any perceived challenges in this clinical work.

Qualitative research to date has generally focused on reasons for malaria over-diagnosis and RDT non-compliance, and has largely been conducted in areas with intense malaria transmission [11–18]. One recent study in the pre-elimination context of Zanzibar specifically investigated how non-malaria fevers are managed in peripheral clinics, and found health workers generally trust negative RDT results but have difficulty differentiating viral from bacterial infections [19]. Similar research is needed from low- to moderate-transmission areas in mainland sub-Saharan Africa where managing non-malaria fevers is common practice.

This paper explores how non-malaria paediatric fevers are managed by health workers at lower level facilities in the low-transmission setting of Mbarara District (Uganda), including RDT perceptions, strategies to differentiate among non-malaria fevers, influences on clinical decisions, desires for additional diagnostics, and challenges faced in this work. Caregivers of children under 5 years old are similarly interviewed about their RDT perceptions and treatment preferences for non-malaria paediatric fevers to check for consistency or disagreement among respondents in order to develop a broader understanding of potential barriers to managing non-malaria paediatric fevers in this setting.

## Methods

### Study site

This study was conducted in Mbarara District, which is a largely rural farming district situated 270 km southwest of Kampala. This district is home to nearly 500,000 people with half the population under 18 years old [20]. Malaria transmission peaks in March–May and September-December, and a reduction in malaria transmission has occurred in recent years [21]. A recent survey found low prevalence (5 %) of malaria infection in young children in the southwestern region of Uganda [22].

There are 58 health facilities in the district (49 government and 9 private) [23]. The first level of the district health system (Health Centre I, or HC-I) includes community-based services delivered by village health teams. The next level includes Health Centre II (HC-II) facilities that provide outpatient services, and are generally led by an enrolled or registered nurse trained to manage common diseases and to provide family planning and antenatal care services. HC-III facilities are generally led by a clinical officer, and are equipped with an outpatient clinic, maternity ward and may have functional laboratory services [24]. Private drug shops are also an important means to obtain medicines by community members [25].

Uganda mainly uses Astel™ or CareStart™ for the detection of histidine-rich protein 2 (HRP2) from *Plasmodium falciparum* [26]. Nationwide RDT deployment was initiated in December 2012, and was accompanied by basic training in RDT use and integrated malaria management in most districts. This training targeted health workers at all levels, including the private sector [27].

### Data collection

A qualitative approach based on in-depth interviews with health workers and focus group discussions (FGDs) with caregivers of children under 5 years old was used in this study. Data collection was carried out during a 3-week period in the dry season (July 2014).

Twenty in-depth interviews and seven FGDs were conducted with sample sizes determined by topical saturation [28]. Health workers interviewed included 13 women and seven men. There were four clinical officers, one midwife, 11 nurses and four nurse assistants, and participants largely worked at lower level public facilities (eight in HC-II and ten in HC-III) except two who worked in HC-IV facilities. Each HC-II facility conducted malaria diagnosis using RDT while higher level facilities generally had both RDT and microscopy capacity. Participant age and work experience ranged from 23 to 58 years and 1.5 to 30 years, respectively. Seven FGDs were also conducted that included caregivers of children under 5 years old living in facility catchment areas. FGD participants included mothers aged 20–43 years old and two grandmothers above 50 years.

For health workers, in-depth interviews each lasting 30–60 min were conducted at a purposive sample of facilities, primarily targeting lower level, government-run, health centres (HC-II, -III) dispersed across the two health sub-districts that were identified in collaboration with the Assistant District Health Officer. In each facility, one health worker responsible for outpatient sick child consultations was asked to participate in the study. Interviews were conducted in English in a private office within the facility by the study authors (EWJ, FEK, CM) with one research assistant to make up two interview teams, each comprised of two people. All interviewers are trained in epidemiology and/or pharmacy. One researcher led the interview while the other recorded observations and any non-verbal communication.

For caregivers, FGDs each lasting 60–90 min were conducted in catchment areas of participating facilities using convenience-sampling techniques. A purposively selected group of caregivers with children under 5 years were identified in collaboration with village leaders. FGDs included 6 to 12 participants and were led by an experienced social scientist fluent in the local language (*Runyankole*) and accompanied by a notetaker to record observations. The moderator received a half-day training on the study’s purpose, interview goals and topic guide. The moderator and study team discussed emerging themes from discussions after each FGD.

Interviews were based on a semi-structured topic guide that focused on how non-malaria paediatric fevers are managed at lower level facilities. For health workers, specific themes included: RDT perceptions; influences on testing and treatment decisions; strategies to differentiate non-malaria paediatric fevers; understandings of potential alternative diagnoses; desires for additional support; and, any dilemmas or challenges. For caregivers, the topic guide explored: RDT experiences and perceptions; understandings of paediatric fever causes; treatment preferences for RDT-negative children; and, acceptability of withholding anti-malarial drugs. Both topic guides were pilot tested and results discussed among the study team. These interviews were included in the analysis since there were few resulting modifications to study tools.

### Data management and analysis

All interviews and FGDs were audio recorded and complemented with any field notes. Recordings were transcribed and translated into English (for FGDs) by bilingual research assistants working on a similar research project in the district. The lead author (EWJ) checked interview transcripts for health workers against the recordings to ensure their accuracy, and FGD transcripts were separately reviewed and cleaned. All transcripts were carefully read multiple times by the lead authors (EWJ, FEK) and were separately coded using a latent content analysis approach [29]. Data were extracted into meaning units that informed an initial coding scheme. Preliminary codes were refined by EWJ and applied back to the transcripts. These codes were discussed and revised by EWJ and FEK, and grouped into mutually agreed themes to describe response patterns. These themes were further refined into a set of final categories that reflected the study objectives and notable ‘clusters of influence’ according to the Diffusion of Innovations theory that is further described in later sections. Briefly, this theory emerged in the 1960s with Everett Rogers seminal work to understand how, why and at what rate new ideas or innovations are adopted within a social group [30]. Since this time, the diffusion of innovations theory has been reviewed, modified and applied across different disciplines, including the spread of innovations within health care organizations [31–34]. Open Code 4.01 (University of Umeå, Sweden) was used for data analysis [35].

### Ethical considerations

Ethical approval was obtained from the WHO Ethical Review Committee, the Makerere University School of Public Health IRB (IRB00011353) and the Uganda National Council for Science and Technology (HS 1385). Individual, written, informed consent to participate in the study and to audio record conversations was obtained from all participants. Prior to involvement, participants were verbally informed about the research purpose and protocols, confidentiality arrangements, and how audio recordings would be handled. All personal information identifying participants was omitted from transcripts.

## Results

Interviews started by asking health workers about perceived common fever causes among sick children visiting their facility. Malaria, pneumonia, flu, common cold, diarrhoea were commonly mentioned in addition to several responses of urinary tract infections, viral infections, otitis media, typhoid, and anaemia. Caregivers similarly responded: malaria, flu, cough, diarrhoea, worms, bad food as well as mention of ear infections, pneumonia, urine problems, skin wounds, enlarged spleen (*ekibaare*), syphilis, typhoid, measles, chicken pox, and evil spirit attacks (*mahembe*).

### Fever differentiation strategies

Most health workers agreed that a negative RDT result prompted them to probe further for alternative causes, while a positive result was not mentioned as eliciting this same clinical decision. Few health workers, however, spontaneously mentioned IMCI as a classification tool for differentiating fevers, although most spoke about taking histories and assessing symptoms as main probing strategies. Symptoms commonly noted as important for illness differentiation included diarrhoea, cough or breathing problems (as indications of pneumonia) as well as skin, ear, eye or urinary infections, anaemia, and dehydration. Studying fever patterns was mentioned, particularly by clinical officers, as important for differential diagnosis. Intermittent fevers were noted to indicate malaria while persistent fevers could be other infections and typhoid may result in a stepladder fever pattern.

*“It depends on the duration and their pattern because we have the stepladder fever, we have the on*-*and*-*off, we have the persistent. The persistent is mainly due to infection. The fever that is on*-*and*-*off is malaria. The stepladder that one could be typhoid but when you get such history you do an exam and you send them for confirmatory diagnostic tests*.” (Health worker 3)

Some health workers spontaneously mentioned analysing other factors for fever differentiation, such as home environment or mosquito net use. Interviewers also explicitly probed for the influence of a child’s age or malaria transmission season on testing and treatment practices. Health workers generally reported testing all suspected cases (e.g., fever) no matter the age or season, although malaria suspicion seemed lower for young infants or in dry seasons. A few health workers responded that they did not test young children under 2 or 4 months old, and these children were either referred or not suspected of having malaria.

Another common strategy was to confirm the negative RDT result with microscopy if available, or to refer for blood smear confirmation at other facilities. One health worker, in contrast, mentioned ordering microscopy for RDT-positive cases to determine illness severity and parasite load.

*“We use [microscopy] when we see that this person has malaria clinically, and have tested using RDT and it tests negative. So you decide to test with the microscope.”* (Health worker 18)

### Influences on managing non-malaria paediatric fevers

Overall, many health workers described prescribing malaria treatment despite a negative RDT result if no alternative cause was found and microscopy confirmation or referral was not possible. Alternative fever causes that could prompt different treatment options (notably antibiotics or oral rehydration solutions) were suspected pneumonia (e.g., cough, difficult breathing) or diarrhoea as well as some mentions of eye, ear, skin or urinary infections.

*“A child comes with fever when it is due to other illnesses like pneumonia, diarrhoea, so you find most of the signs are much related with other conditions and not malaria. So we don’t usually consider that fever to be malaria. But sometimes if the fever is not associated with other signs and symptoms of other conditions so there you put a question mark and you go ahead and treat [for malaria]”* (Health worker 1)

Malaria over-treatment for RDT negative results also seemed driven by a combination of RDT perceptions, system constraints and provider-client interactions that in turn elicited various dilemmas or feelings in health workers about managing non-malaria paediatric fevers.

### RDT perceptions

Health workers expressed general RDT mistrust that was either explicitly mentioned during interviews or implied by a stated preference to confirm the negative RDT result using microscopy.

*“You see many signs and symptoms like fever, headache and so on and yet the results of the RDT show otherwise. Me I don’t usually trust the results of the RDT.”* (Health worker 15)

In many cases RDT doubts were fueled by previous experience where multiple testing gave contradictory results (e.g., RDT negative but blood smear positive). One health worker, in contrast, doubted the RDT positive result while two others expressed strong trust in RDT results, even over blood smear readings.

*“We have come to realize there is no way I can prove that [RDTs] are not working or they are working because sometimes you test a person and she is negative. But clinically you really see that is malaria and then usually I wonder there are some cases you put on RDTs and they become negative but when you put on microscope, positive.” (Health worker 1)*

This general RDT mistrust also seemed driven by expectations of false negative results in certain situations, including low parasite/antigen loads, previous anti-malarial dose or test detection of only one species. Table 1 highlights the various ways these perceptions were expressed to interviewers.

**Table 1:** Rapid diagnostic test perceptions and managing non-malaria paediatric fevers

Low parasite/antigen load	Previous anti-malarial dose	Test detection of only one species
*But mostly we prefer the high temperature for about like 2* *days. So we decide [to test using RDT].* (Health worker 13)	*She comes here and she tells you that she has already taken 4 tabs [of malaria treatment] from somewhere. So you get a sample from her and you do an RDT. The RDT can come negative. But since she has already told you that she has taken the treatment, you give her treatment to finish the course*. (Health worker 13)	*I think if it’s some other* Plasmodium *it could be malaria but not detected using the RDT.* (Health worker 5*)*
*For RDTs it is rare to confirm malaria when the parasites [sic] have just developed fever. But if somebody has been down and has not been getting treatment there you can confirm [using RDT].* (Health worker 14)	*In most cases you find when a patient is taking anti*-*malarials you test there is no fever, I mean there is no malaria. I think you take the patient to another step or second drug, second choice of drug or you refer to health centre IV for management.* (Health worker 19)	*The RDT tests only for one species of malaria. The person could be having other types of malaria and when you give the treatment, although the result was negative, the person responds.* (Health worker 8)
*Malaria is still starting and has not yet spread out. So it is difficult to detect, for example, if somebody contracted malaria last night and you test today, it will be hard for you.* (Health worker 14)	*When the child has not taken any anti*-*malarial, and has taken like 3*–*4* *days, and the blood slide is negative there we totally don’t treat malaria.* (Health worker 4)	
*Sometimes when the malaria parasites are hidden you give an anti*-*malarial, and some do improve.* (Health worker 15)	*When they have taken coartem from the health centres or from the drug shops anywhere, so there you ask if she has taken any treatment. Then if it’s there you give the second line.* (Health worker 4)	
*I think these RDTs to become positive that malaria might have persisted or it might have taken like 3* *days. But the malaria of 1* *day cannot be positive on an RDT. It can’t.* (Health worker 18)	*We take the history, if there is a fever and some other presenting complaints maybe there has been headache, vomiting, then also if the child has not been on anti*-*malarial treatment before we test using RDT. But if he has been on anti*-*malarials before, we go on for microscopy.* (Health worker 5)	
*When she spent there like 3* *days is when you get the reaction immediately after testing using the RDTs. But sometimes when that person got fever at that moment and comes for RDTs we don’t get immediate results from the malaria parasites, so we advise that person maybe we treat that person clinically.* (Health worker 2)	*Like someone may say I took some anti*-*malarials a week before. No improvement. My child still has fever. You do RDT and it tests negative but clinically you see this child might be having fever. So we go ahead and do microscopy.* (Health worker 9)	
*When somebody comes and you give the first test immediately and you cannot get positive response. But if someone is sick for 3 or 4* *days back you get the positive results.* (Health worker 2*)*	*Sometimes when someone has taken anti*-*malarials it is hard for an RDT to test positive.* (Health worker 9)	
*When the child has not taken any anti*-*malarial, and has taken like 3*–*4* *days, and the blood slide is negative, there we totally don’t treat malaria.* (Health worker 4)	*But also what helps to diagnose malaria if the RDT is negative, some caretakers give anti*-*malarials like days before they come. But it is always not a complete dose. The RDT will tell us negative. But if the caregiver tells us the truth that I gave anti*-*malarials and I didn’t complete the dose, we start afresh.* (Health worker 5)	
*Because of the incubation period. If the incubation period has not ended then we treat [for malaria].* (Health worker 4)	*Some of us go to clinics first and we are given medicine without testing. So the malaria parasites hide. But somehow when they go to test elsewhere the malaria is not detected but the doctor kind of understands this and goes ahead and treats malaria.* (FGD 1 participant)	
*You may find the child having fever for 2* *days. That one I can easily go and RDT test.* (Health worker 11)		
*I took my child and they tested him, and they detected few malaria parasites, so they gave treatment for malaria because the malaria parasites would eventually increase if left untreated and cause serious malaria.* (FGD 2 participant)		
*What happens is that you see a child has high temperature, you may doubt. Even if they say it is not there, but it could rise so you buy medicine in preparation*. (FGD 5 participant)		
*This is what they say, they say they have a gadget they use to test and say that the malaria is not yet in the blood. It is still in the lungs [sic], and so they give you anti*-*malarial drugs.* (FGD 7 participant)		

### Low parasite/antigen loads

First, many health workers explained that an RDT positive result is difficult to obtain during initial illness stages since RDTs may be insensitive to low parasite/antigen loads. In these cases, malaria treatment for an RDT-negative result was generally seen as appropriate in order to treat the malaria infection the RDT could not yet detect. Some health workers expressed this phenomenon as a parasite ‘incubation period’ or that the parasites are ‘hidden’, ‘have not yet matured’ or ‘have not yet spread out’ (Table 1). Some health workers reported this period could last about 3 days from the start of the fever episode. Several caregivers similarly described their experience of receiving malaria treatment for their RDT-negative child because *‘the malaria is not yet in the blood’* (FGD 7 participant).

*“I think that these RDTs to become positive that malaria might have persisted or it might have taken like three days. But the malaria of one day cannot be positive on an RDT. It can’t.”* (Health worker 18)

### Previous anti-malarial dose

Second, many health workers mentioned prescribing malaria treatment for an RDT negative result if the child had recently taken an anti-malarial dose. There were two distinct reasons for this practice. Some respondents suggested a need to complete the treatment course even if the patient had no detectable malaria infection.

*“Sometimes a patient comes with a history of fever and has already taken some medication at home. She comes here and she tells you that she has already taken four tabs [of malaria treatment] from somewhere. So you get a sample from her and you do an RDT. The RDT can come negative. But since she has already told you she has taken the treatment, you give her treatment to finish the course.”* (Health worker 13)

A few health workers also stated that a positive result could be difficult to obtain if an anti-malarial drug was already present in a child’s system. One caregiver related this experience during the FGD.

*“Some of us go to clinics first and we are given medicine without testing. So the malaria parasites hide. But somehow when they go to test elsewhere the malaria is not detected but the doctor kind of understands this and goes ahead and treats malaria.”* (FGD 1 participant)

### Test detection of only one species

Third, in a few instances, health workers mentioned that the RDT detects only one parasite species and malaria infection may not be detected if caused by another agent.

*“The RDT tests only for one species of malaria. The person could be having other types of malaria and when you give the treatment, although the result was negative, the person responds.”* (Health worker 8)

### System constraints

Many health workers also mentioned health system constraints that fueled malaria over-treatment, notably poor referral systems, working alone without opportunity to confer on difficult cases, and lacking skills and/or tools for differential diagnosis. These interconnected system constraints were clearly expressed as follows:

*“You can see the result is negative. The child is seriously sick. When you talk of referral, the mother is there complaining. Then you are there, and you say: ‘Now what? What can I do?’ But if we could be equipped well with other things. You can do a test and it proves the cause of the sickness or if you have other cadres of human resources, they can do it. There is no doctor. You are there. You are alone. So at least if you are a nurse and you fail on something, you can consult a doctor or a nursing officer. There is nobody.”* (Health worker 8)

Referral was a commonly mentioned challenge and many HC-II nurses expressed a desire to refer RDT-negative cases for microscopy confirmation or doctor’s care since they felt unable to manage these cases themselves. Yet referral challenges limited this option.

*“Yes it is a challenge! Now what do you think we can do with those patients that test negative? What do you think we can do? And they don’t have any other causes of fever. We do what, we refer. Yes you refer. It is difficult because you tell them refer but the mother has no transport. It’s a problem.”* (Health worker 18)

Several respondents mentioned inadequate skills and/or tools for differential diagnosis, and these problems seemed compounded by working alone without opportunity to confer on difficult cases.

*“Other conditions which cause fever, we don’t have the test. We just do physical examinations. Sometimes like lymph nodes we know the person has a bacterial infection. Yes it is a challenge. But sometimes we sit in the village, we forget. We need refresher training for the health workers about how to manage those conditions.”* (Health worker 12)

*“Sometimes we have been asking ourselves what happens to the RDTs. What brings that? As I have told you that I am a nursing assistant. So I start asking myself what can I do. Maybe I think about if I am with a clinical officer but on my own, according to how I can manage, if I can’t manage I refer.”* (Health worker 13)

Inadequate supplies of RDTs and essential medicines were also commonly mentioned problems. Interviewers specifically probed about other tools that could help manage non-malaria fevers. Responses included thermometers, stethoscopes, pulse oximeters, respiratory rate counters, urine analysis, weighing scales, typhoid and brucella tests, although each item was mentioned only once or less than a few times. Importantly, some HC-II nurses specifically desired microscopes for malaria diagnosis. Caregivers also noted these same system constraints, and commonly expressed desires to attend ‘bigger hospitals’ or to visit doctors seen as more capable of differential diagnosis.

*“When you reach the hospital there are many tests they can conduct. They could test for malaria, typhoid. So if I suspect malaria, the doctor should decide what else could be bothering the child.”* (FGD 1 participant)

### Provider-client interactions

The provider-client interaction also influenced the management of non-malaria paediatric fevers, including caregiver RDT mistrust, demand for certain drugs, and desire to know the ‘exact’ disease cause if not malaria.

Many health workers perceived caregivers as lacking malaria knowledge, although a few noted that caregivers increasingly understood that all fevers are not due to malaria. This was attributed to the remaining stigma from previous presumptive treatment policies. Yet, FGDs showed a proven awareness among caregivers of other fever causes. Most health workers felt that while caregivers accepted malaria diagnosis, or were even eager for testing, there remained some mistrust of negative results. This overall perception was also reflected in caregiver responses.

*“By the way the caregivers like those blood tests very much. And when you do it they are contented. Although those with fever, they still feel that the test was not perfect even if you tell them that there is not malaria. But they like it.”* (Health worker 15)

Many health workers also complained that caregivers demanded malaria treatment and felt health workers *must* treat the sick child. Similarly, most caregivers said they wanted to receive treatment to cure the child’s sickness. Some would demand malaria treatment if they strongly felt that was the fever cause, although others talked about accepting the result and following the clinical decision. In general, caregivers wanted to know the ‘exact’ disease cause if not malaria.

*“So some parents go ahead and ask for anti*-*malarial drugs. Some hospitals give into the parents who insist on getting the drugs.”* (FGD 7 participant)

*“My thinking would be that if they don’t detect malaria then they should be able to detect any other diseases. If they don’t say anything else I would go to another hospital with testing machines and get to know what the child is suffering from.”* (FGD 2 participant)

### Dilemmas and feelings

The challenges described above elicited various dilemmas or feelings about managing non-malaria paediatric fevers. Many health workers expressed uncertainty about how to manage non-malaria fevers; feared doing wrong, loss to follow-up or patient death; worried caregivers would lose trust; or, felt unsatisfied without a clear diagnosis.

Some health workers seemed unsure about how to manage non-malaria fevers, which was either explicitly mentioned during interviews or implied by asking interviewers for guidance.

*“Maybe I don’t understand very well those RDTs because sometimes you see someone who is really sick. But you test the RDT and it is negative. But when you give anti*-*malarial treatment that patient improves and becomes okay. I don’t know what advice you can give us on such patients. Should we continue giving them anti*-*malarials or we refer them for microscopy?”* (Health worker 12)

Some also expressed a desire to consult with doctors given uncertainty about managing RDT-negative cases. Many caregivers similarly expressed greater trust in doctors to manage non-malaria fevers, as previously described.

*“What I know is that if it’s a more qualified doctor, he checks ears, urine and you find maybe the ear has an infection and you didn’t know.”* (FGD 5 participant)

Some health workers also feared doing wrong, patient loss to follow-up or patient death, which was clearly expressed as follows:

*“We know malaria is a killer. It actually kills more people than accidents. So if you left this child and the child went back home, especially those young ones below five years, you are not sure whether the parents are going to continue to both assess and monitor the status of this child. So in case you miss out, we fear maybe this child is going to die before they come back. So you would rather give treatment than leave this child to go home because some of them come from very far. Others do not even have money maybe to rush to the nearest clinic in case things happen when it is late. So you know, you put all those things into consideration.”* (Health worker 10)

Several health workers also worried that caregivers would lose trust in their services, particularly if clients strongly believed the RDT-negative patient had malaria and there was no alternative diagnosis. Again, caregivers expressed less trust in peripheral clinics to manage non-malaria fevers underscoring such concerns.

*“[Clients] lose trust in you because you’ve told them it’s not the disease when they know it’s the disease.”* (Health worker 20)

*“These hospitals to me they are good because they have skilled professionals, sometimes when you go to a nurse they may not detect the disease.”* (FGD 1 participant)

A few health workers also felt unsatisfied without a clear diagnosis, desired to do a better job, and expressed dissatisfaction to *‘just blindly treat to see what will come out’* (Health worker 15).

*“Sometimes when somebody has fever and the RDT is negative, and they don’t have pneumonia and they just have fever, it is quite challenging because we don’t know the cause of the fever. You could think it could be viral but you don’t know, you have not diagnosed it. So we just give antipyretics. But inside you, you are not satisfied. You feel you would have done better but you can’t. You don’t have anyway how to do it.”* (Health worker 5)

Many caregivers shared this desire to know the ‘*exact disease’* causing the child’s illness.

*“You want to make sure you know the exact disease. It is like the reason why you take the child to hospital is to know the exact disease. Because one may as well go to the drug shop to buy drugs.”* (FGD 5 participant)

Health workers generally responded to these dilemmas by justifying their clinical decisions, and by noting that RDT-negative patients often improve on malaria treatment. Interviewers also asked about the potential downsides of malaria over-treatment. Several health workers noted increasing drug resistance and incorrectly treating other diseases, while a few mentioned wasting resources and burdening a child’s body with drugs to eliminate. One health worker said there were no downsides while a few were unsure how to respond.

## Discussion

Study findings indicate that malaria over-treatment for RDT-negative results may occur in this low-transmission setting if no alternative fever cause is identified. RDT non-compliance seemed further driven by a combination of RDT perceptions, provider-client interactions and system constraints that must be addressed to improve rational drug use and quality fever management at lower level clinics in Mbarara District, Uganda.

Importantly, these constraints reflect long-established clusters of influence on the spread of new innovations or practices according to the Diffusion of Innovations theory [30–34], which was recently adapted to the RDT experience in sub-Saharan Africa [13].

RDT perceptions included a general RDT mistrust among health workers and caregivers, which has been described in other settings [11, 13–18]. This mistrust developed in part from provider experiences with contradictory results between RDT and blood smear readings, and also from perceptions that a positive result is difficult to obtain for certain reasons: low parasite/antigen loads, previous anti-malarial treatment and test detection of only one species. Many health workers raised concerns about RDT insensitivities to low antigen concentrations, which is more likely to occur at early illness stages [36]. Many health workers also discussed RDT insensitivities if a child recently took an anti-malarial dose, although current evidence does not support this assertion. Others desired to complete the treatment dose despite a negative result so as not to create resistance ‘in a child’, but such concerns are unwarranted if malaria parasites are not present [37]. A few health workers mentioned that RDTs could not detect malaria if the infection is caused by a species other than *P. falciparum*.

These findings show a proven awareness of potential problems with RDT malaria detection, such as for low parasitaemias or to detect certain parasite species [36], and legitimate concerns regarding anti-malarial drug resistance [37]. Yet this awareness has been misconstrued to inappropriately justify RDT non-compliance, and to support a general preference to diagnose malaria using routine microscopy or to confirm RDT negative results with blood smears. This preference is reinforced by national guidelines that promote microscopy as the ‘gold standard’ for malaria diagnosis in Uganda [3]. It may also reflect an underlying desire to continue presumptive treatment practices, a long-standing policy that is generally easier for health workers to implement.

Yet, current evidence shows RDTs are sufficient to clinically manage suspected cases in low-transmission settings with equal or better performance than routine microscopy [38], and low quality routine microscopy for malaria has been previously documented [39]. Moreover, these understandings do not excuse a lack of probing for other fever causes, particularly for patients with early illness symptoms where some health workers seemed to expect false negative results up to 3 days after fever onset. Such practices could unnecessarily delay appropriate fever management with potentially fatal consequences [40].

The Diffusion of Innovations literature suggests that innovation perceptions may explain a large part of the variance in the adoption of new practices [30, 34], and five innovation perceptions are most influential for successful uptake: benefit, compatibility, simplicity, trialability, and observability.

First, users need to see a relative *benefit* to the status quo if RDTs are adopted, which includes reducing any perceived risks in employing the new innovation or practice. While most health workers understood the advantages of malaria diagnosis and caregivers seemed eager for testing, most respondents perceived inherent ‘risks’ in the new practice of managing RDT-negative patients—notably missing a malaria diagnosis—that may greatly reduce any perceived benefits. This perception of missing a malaria diagnosis as ‘risky’ is consistent with other research [12]. These perceived risks could be addressed through messaging that focuses on the reliability of RDT-negative results [38], the demonstrated safety of withholding anti-malarial treatment [41], and the deliberate over-treatment built into the IMCI algorithm for other fatal febrile illnesses (e.g., bacterial pneumonias, measles, diarrhoeal diseases) in order to specifically avoid severe consequences in patients [1]. This could help build trust in negative results, and reduce perceived risks in managing non-malaria paediatric fevers if RDT and IMCI are correctly implemented together.

Second, RDT implementation needs to be *compatible* with current clinical practice. In this study, however, most health workers did not find their current training or available tools compatible with the new practice of managing RDT-negative patients. Third, *simple* technologies are often more readily adopted than complex ones. While RDTs are simple to use, RDT-negative patients were generally perceived as complicated to manage. Finally, *trialability* (having a trial or testing period) and *observability* (watching others use the innovation or employ the new practice) also aids adoption. Trialability and observability, in particular, are especially important for adopting ‘risky’ practices in order to give users space to experiment with the new practice and to understand how others have incorporated it into their own work. This provides critical, early opportunities to share experiences, answer questions, give feedback, address concerns, adapt the new practice to routine work, and build confidence that others are working in a similar manner [34].

Provider-client interactions emerged as factors influencing management of non-malaria paediatric fevers, which have been highlighted elsewhere [16, 18]. Many health workers perceived clients as lacking malaria knowledge and demanding certain medicines. Yet these perceptions were not necessarily supported by FGD findings. Overall, caregivers had a proven awareness of other potential fever causes in children. While some mistrusted RDTs and might demand anti-malarial medicines, many also seemed willing to accept a negative test result but desired an alternative diagnosis to understand their child’s sickness. To this end, many caregivers preferred visiting doctors or higher level facilities, seen as better equipped to identify and treat the *exact* disease cause.

Indeed, the Diffusion of Innovations theory highlights user characteristics as another sphere of influence on innovation adoption, which in this context includes both providers and clients. Some authors have categorized users as ‘innovators’, ‘early adopters’, ‘early majority’, ‘late majority’, and ‘laggards’, and these categorizations may also pertain to adopters in service organizations [30, 34]. Early adopters may be seen as opinion leaders characterized as professionally respected and resourceful. Evidence points to the critical role of opinion leaders in promoting innovation adoption by shaping peer opinion [30–34]. In this district, some clinical officers could naturally fit that role but greater investments would be needed to build up this network, and to subsequently connect such ‘opinion leaders’ with other health workers in the district. Clinical officers seemed more knowledgeable than lower health worker cadres regarding paediatric fever causes and their management, which was a perception shared by most caregivers too. In fact, both nurses and caregivers interviewed expressed a clear desire to consult with doctors in order to manage non-malaria paediatric fevers.

Moreover, evidence suggests that organizations that foster informal exchanges among users may experience faster rates of practice adoption [30–34], and some research has also demonstrated improved health outcomes by facilitating such interactions [42]. Along these lines, recent studies have shown improved RDT compliance using interactive training programmes or daily SMS reminders that essentially form a basis for improved communication networks and connectedness among adopters [43, 44]. Other researchers have also recommended establishing communities of practice, Balint groups or peer-learning networks to increase problem-solving and mentorship opportunities to improve RDT compliance [13]. One additional intervention could include providing airtime to nurses at lower level clinics to facilitate real-time consultations with opinion leaders on difficult cases, such as RDT-negative patients.

Clients are also ‘users’ in this context and play a role in determining care-seeking strategies and shaping the clinical encounter [45]. Community opinion leaders are therefore needed to help raise awareness about new clinical practices and build community trust in negative results. To date, community sensitization to new clinical practices in this district has largely relied on health worker counselling of patients, despite their limited time. While there is a need to strengthen health worker counselling skills, FGD findings also suggest that health workers may not be suitable opinion leaders within communities, given some negative perceptions expressed by caregivers.

System constraints included poor referral systems, working alone without opportunity to confer on difficult cases, and lacking skills and/or tools for differential diagnosis, which are well-known issues in Uganda [46]. Few health workers specifically mentioned using IMCI guidelines to differentiate among fever causes, which suggests a lack of awareness of the primary tool available to classify non-malaria fevers. Some health workers referred to aspects of this tool but none described employing it consistently or even correctly in their practice, and poor IMCI implementation has previously been shown in Uganda and other settings [47, 48].

It is critical that lower level health workers feel empowered to manage non-severe, non-malaria paediatric fever cases without referral, which is the opposite of study findings. There is general consensus about the need to deploy RDT as part of integrated fever management protocols, notably IMCI for sick children [10]. IMCI has been shown to improve quality of care for common causes of child morbidity and mortality (e.g., malaria, pneumonia, diarrhoea, measles, malnutrition, and ear infections), which often include fever as a presenting complaint [49]. There are also ongoing initiatives to strengthen the IMCI algorithm based on recent etiology studies [50, 51], and in recognition of its poor implementation to date [48]. Effective implementation of IMCI and RDT together could help reduce both anti-malarial and antibiotic over-treatment associated with RDT-negative results shown in other studies [5–9], and suggested by findings for this district. Moreover, integrated community case management (iCCM) using a simplified IMCI algorithm and RDT has been successfully implemented by community health workers and drug shop attendants in research studies [52, 53]. Yet, if these pilot studies are more broadly implemented, these community volunteers would be supervised by lower level health workers that remarkably lack similar training in integrated care, as suggested in this study.

This lack of IMCI skills to manage non-malaria fevers is further compounded by inadequate tools or other diagnostics to differentiate fevers, poor referral systems and feelings of working alone without opportunities to consult on difficult cases, which have been discussed elsewhere [54]. Developing communities of practice and fostering exchanges among health workers, as previously described, could potentially reduce feelings of working alone without support, reduce the desire to refer non-severe cases, build trust in nurses ability to handle non-malaria fevers, and satisfy caregiver desire to attend bigger hospitals by better linking doctors to peripheral clinics. Nevertheless, current findings suggest that some over-treatment of dangerous illnesses will remain the norm in settings with weak health systems where providers fear a child may not return if symptoms worsen, and are unable to refer them for further testing and medical care.

### Methodological considerations

The qualitative design was chosen to understand how health workers manage non-malaria paediatric fevers at lower level clinics and to elucidate reasons for such practices, but findings reflect only *stated* rather than *observed* actions. These stated preferences could reflect desires to please interviewers [28]. Yet, triangulation of data collected across different interview teams, among different health worker cadres and with FGDs, indicates broad consistency in information derived from various respondents. The geographical spread of interviews across numerous lower level clinics in the district provides a good indication of how non-malaria fevers are reportedly managed in this setting, but is not statistically representative. External validity of study conclusions is strengthened by linking to similar findings in other settings, as well as to theoretical constructs that underpin reported phenomena, notably the Diffusion of Innovations theory [28]. Finally, this paper reflects reported practices in managing non-malaria paediatric fevers about 1.5 years after RDT initiation in this district, and may be considered an early assessment that highlights perceptions and practices that need to be addressed going forward.

## Conclusions

Study findings indicate that malaria over-treatment for RDT-negative results reportedly occurs in this setting if no alternative fever cause is found, and RDT non-compliance is further driven by a combination of RDT perceptions, system constraints and patient-client interactions. Enhanced support is needed to improve RDT compliance at lower level clinics that empowers health workers to successfully manage non-severe, non-malaria, paediatric fevers without referral. This includes building trust in negative results, reinforcing skills in integrated fever care, and facilitating Communities of Practice according to the Diffusion of Innovations theory. Such interventions could not only improve RDT compliance, rational drug use and quality fever care, but also strengthen overall health systems with RDT as the entry point.

## Abbreviations

IMCI: integrated management of childhood illness
iCCM: integrated community case management
FGD: focus group discussion
RDT: malaria rapid diagnostic test
WHO: World Health Organization

## References

[1] World Health Organization and United Nations Children’s Fund (1997). Integrated management of childhood illness: a WHO/UNICEF initiative. Bull World Health Organ.

[2] WHO. Guidelines for the treatment of malaria. Geneva: World Health Organization; 2010. 25473692

[3] Government of Uganda, Ministry of Health. Uganda National Malaria Strategic Plan 2014/15. Kampala: Government of Uganda; 2013.

[4] WHO. T3: Test. Treat. Track. Scaling up diagnostic testing, treatment and surveillance for malaria. Geneva: World Health Organization; 2012.

[5] Bastiaens GJH, Bousema T, Leslie T (2014). Scale-up of malaria rapid diagnostic tests and artemisinin-based combination therapy: challenges and perspectives in sub-Saharan Africa. PLoS Med.

[6] Keating J, Finn T, Eisele TP, Dery G, Biney E, Kedote M (2014). An assessment of malaria diagnostic capacity and quality in Ghana and the Republic of Benin. Trans R Soc Trop Med Hyg.

[7] Mubi M, Kakoko D, Ngasala B, Premji Z, Peterson S, Björkman A (2013). Malaria diagnosis and treatment practices following introduction of rapid diagnostic tests in Kibaha District, Coast Region. Tanzania. Malar J.

[8] D’Acremont V, Kahama-Maro J, Swai N, Mtasiwa D, Genton B, Lengeler C (2011). Reduction of anti-malarial consumption after rapid diagnostic tests implementation in Dar Es Salaam: a before-after and cluster randomized controlled study. Malar J.

[9] Msellem MI, Mårtensson A, Rotllant G, Bhattarai A, Strömberg J, Kahigwa E (2009). Influence of rapid malaria diagnostic tests on treatment and health outcome in fever patients, Zanzibar: a crossover validation study. PLoS Med.

[10] WHO. Informal consultation on fever management in peripheral health care settings—a global review of evidence and practices. Geneva: World Health Organization; 2013.

[11] Assimwe C, Kyabayinze DJ, Kyalisiima Z, Nabakooza J, Bajabaite M, Counihan H (2012). Early experiences on the feasibility, acceptability, and use of malaria rapid diagnostic tests at peripheral health centres in Uganda—insights into some barriers and facilitators. Implement Sci.

[12] Chandler C, Jones C, Boniface G, Juma K, Reyburn H, Whitty CJM (2008). Guidelines and mindlines: why do clinical staff over-diagnose malaria in Tanzania? A qualitative study. Malar J.

[13] Chandler CIR, Whitty CJM, Ansah EK (2010). How can malaria rapid diagnostic tests achieve their potential? A qualitative study of a trial at health facilities in Ghana. Malar J.

[14] Chandler CIR, Mangham L, Njei AN, Njei AN, Achonduh O, Mbacham WF (2012). ‘As a clinician you are not managing lab results, you are managing the patient’: how the enactment of malaria at health facilities in Cameroon compares with new WHO guidelines for the use of malaria tests. Soc Sci Med.

[15] Uzochukwu BS, Onwujekwe E, Ezuma NN, Exeoke OP, Ajuba MO, Sibeudu FT (2011). Improving rational treatment of malaria: perceptions and influence of RDTs on prescribing behavior of health workers in southeast Nigeria. PLoS ONE.

[16] Ezeoke OP, Ezumah NN, Chandler CC, Mangham-Jeffries LJ, Onwujekwe OE, Wiseman V (2012). Exploring health providers’ and community perceptions and experiences with malaria tests in South-East Nigeria: a critical step towards appropriate treatment. Malar J.

[17] Ughasoro MD, Okoli CC, Uzochukwu BS (2013). Qualitative study of presumptive treatment of childhood malaria in third-tier tertiary hospitals in southeast Nigeria: a focus group and in-depth study. Malar J.

[18] Diggle E, Asgary R, Gore-Langton G, Nahashon E, Mungai J, Harrison R (2014). Perceptions of malaria and acceptance of rapid diagnostic tests and related treatment practices among community members and health care providers in Greater Garissa, North Eastern Province. Kenya. Malar J.

[19] Baltzell K, Elfving K, Shakely D, Ali AS, Msellem MI, Gulati S (2013). Febrile illness management in children under 5 years of age: a pilot study on primary health care workers’ practices in Zanzibar. Malar J.

[20] Mbarara District Local Government. http://www.mbarara.go.ug.

[21] De Beaudrap P, Nabasumba C, Turyakira E, Schramm B, Boum Y, Etard JF (2011). Heterogeneous decrease in malaria prevalence in children over a six-year period in south-western Uganda. Malar J.

[22] Government of Uganda, Bureau of Statistics and Ministry of Health. Malaria Indictor Survey 2014–2015—key indicators. Kampala: Government of Uganda; 2015.

[23] Government of Uganda, Bureau of Statistics. National Population and Housing Census 2014. Kampala: Government of Uganda; 2015.

[24] Government of Uganda, Ministry of Health. Health Sector Strategic Plan 2010/11–2014/15. Kampala: Government of Uganda; 2015.

[25] Kitutu FE, Mayora C, Awor P, Forsberg B, Peterson S, Wamani H (2014). Inclusion of private sector in district health systems: case study of private drug shops implementing the modified integrated community case management (iCCM) strategy in rural Uganda. BMC Health Serv Res.

[26] WHO, Foundation for Innovative New Diagnostics, US Centers for Diseases Control and Prevention. Malaria rapid diagnostic test performance: summary results of WHO product testing of malaria RDTs: Round 1–5 (2008-2013). Geneva: World Health Organization; 2014.

[27] Government of Uganda, Ministry of Health. The Uganda Malaria Reduction Strategic Plan 2014–2020. Kampala: Government of Uganda; 2014.

[28] Pope C, Mays N (2006). Qualitative research in health care.

[29] Graneheim UH, Lundman B (2004). Qualitative content analysis in nursing research: concepts, procedures and measures to achieve trustworthiness. Nurse Educ Today.

[30] Rogers EM (1983). Diffusion of innovations.

[31] Greenhalgh T, Robert G, MacFarlane F, Bate P, Kyriakidou O (2004). Diffusion of innovations in service organizations: systematic review and recommendations. Milbank Q.

[32] Institute for the Future (2002). Diffusion of innovations in health care.

[33] Dearing JW (2009). Applying diffusion of innovations theory to intervention development. Res Soc Work Pract.

[34] Berwick DM (2003). Disseminating innovations in health care. JAMA.

[35] University of Umeå (Sweden) ICT Services and System Development and Division of Epidemiology and Global Health. OpenCode 4.01. http://www.phmed.umu.se/enheter/epidemiologi/forskning/open-code/.

[36] Miller E, Sikes HD (2015). Addressing barriers to the development and adoption of rapid diagnostic tests in global health. Nanobiomedicine.

[37] WHO. Global report on anti-malarial drug efficacy and drug resistance: 2000–2010. Geneva: World Health Organization; 2010.

[38] WHO Malaria Policy Advisory Committee2014. WHO evidence review group on malaria diagnosis in low transmission settings—meeting report, 16-18 December 2013. Geneva: World Health Organization; 2014.

[39] Kahama-Maro J, D’Acremont V, Mtasiwa D, Genton B, Lengeler C (2011). Low quality of routine microscopy for malaria at different levels of the health system in Dar Es Salaam. Malar J.

[40] Hildenwall H, Tomson G, Kaija J, Pariyo G, Peterson S (2008). I never had the money for blood testing—Caretakers’ experiences of care-seeking for fatal childhood fevers in rural Uganda—a mixed-methods study. BMC Int Health Hum Rights.

[41] D’Acremont V, Malila A, Swai N, Tilya R, Kahama-Maro J, Lengeler C (2010). Withholding anti-malarials in febrile children who have a negative result for a rapid diagnostic test. Clin Infect Dis.

[42] Persson LÅ, Nga NT, Målqvist M, Hoa DTP, Eriksson L, Wallin L (2013). Effect of facilitation of local maternal-and-newborn stakeholder groups on neonatal mortality: cluster-randomized controlled trial. PLoS Med.

[43] Modrek S, Schatzin E, De La Cruz A, Isiguzo C, Nwokolo E, Anyanti J (2014). SMS messages increase adherence to rapid diagnostic test results among malaria patients: results from a pilot study in Nigeria. Malar J.

[44] Mbacham WF, Mangham-Jeffries L, Cundill B, Achonduh OA, Chandler CIR, Ambebila JN (2014). Basic or enhanced clinician training to improve adherence to malaria treatment guidelines: a cluster-randomized trial in two areas of Cameroon. Lancet Glob Health.

[45] Orem N, Mugisha F, Okui AP, Musango L, Kirigia JM (2013). Health care seeking patterns and determinants of out-of-pocket expenditure for malaria for children under five in Uganda. Malar J.

[46] Uganda Ministry of Health, Makerere University School of Public Health. Uganda health system assessment 2011. Kampala and Bethesda (MD), Health Systems 20/20 Project, Abt Associates Inc.; 2012.

[47] Pariyo GW, Gouws E, Bryce J, Burnham G, The Uganda IMCI impact study team (2005). Improving facility-based care for sick children in Uganda: training is not enough. Health Policy Plan.

[48] Chopra M, Binkin NJ, Mason E, Wolfheim C (2012). Integrated management of childhood illness: what have we learned and how can it be improved?. Arch Dis Child.

[49] Amaral J, Victora CG (2008). The effect of training in integrated management of childhood Illness (IMCI) on the performance and healthcare quality of pediatric healthcare workers: a systematic review. Revista Brasileira de Saude Materno Infantil.

[50] D’Acremont V, Kilowoko M, Kyungu E, Philipina S, Sangu W, Kahama-Maro J (2014). Beyond malaria—causes of fever in outpatient children. NEJM.

[51] Hildenwall H, Amos B, Mtove G, Muro F, Cederlund K, Reyburn H (2015). Causes of non-malarial febrile illness in outpatients in Tanzania. Trop Med Int Health.

[52] Mukanga D, Tiono AB, Anyorigiya T, Källander K, Konate AT (2012). Integrated community case management of fever in children under five using rapid diagnostic tests and respiratory rate counting: a multi-country cluster randomized trial. Am J Trop Med Hyg.

[53] Awor P, Wamani H, Peterson S (2015). Drug seller adherence to clinical protocols with integrated management of malaria, pneumonia and diarrhoea at drug shops in Uganda. Malar J.

[54] Iwelunmor J, Airhihenbuwa CO, King G, Adedokun A (2013). Contextualizing child malaria diagnosis and treatment practices at an outpatient clinic in southwest Nigeria: a qualitative study. ISRN Infec Dis.

